# Efficacy of nutritional treatment in patients with psoriasis: A case report

**DOI:** 10.3892/etm.2019.8298

**Published:** 2019-12-05

**Authors:** Ang Peng Wong, Tatiana Kalinovsky, Aleksandra Niedzwiecki, Matthias Rath

Exp Ther Med 10: 1071-1073, 2015; DOI: 10.3892/etm.2015.2631

We received an email from a concerned reader regarding the claims made in the above paper that a patient experienced resolution of psoriatic patches on her body after having received a cocktail of nutritional supplements, while adhering to a regimen that prescribed dietary changes for a period of 6 months. Although the findings, while preliminary in nature, were potentially encouraging, the Editor of *Experimental and Therapeutic Medicine* agrees with the reader that the study exhibited a number of limitations. A follow-up study was not conducted, and the possibility of disease recurrence was not investigated. A follow-up study of the patient examined would also have demonstrated the effectiveness of this therapy in a long-term period. Moreover, a further limitation is that, since the present study was a case report, the findings reflect the response of an isolated patient incident. Clinical studies with a larger sample size would offer clearer insights into the efficacy of this therapy, allowing the statistical determination of clinical outcomes and benefits. Finally, the Editor also noted that the study contained an undeclared potential conflict of interest, since the nutritional supplements Vitacor Plus, ProLysinC, VitaCforte and LysinC Drink mix are sold by a company owned by Stichting Administratiekantoor Dr. Rath Holding, to whose companies group the sponsor of the study also belongs, and therefore there are commercial and financial implications associated with this case report.

Based on the decision of the Editor, we wish to publish this corrigendum to highlight these issues. Furthermore, the limitations detailed above have been added to the online version of the paper as a note added post-publication for the printed record, and a competing interest statement concerning the potential conflict of interest has also been added to the Declarations section. Note that the following authors (Aleksandra Niedzwiecki and Matthias Rath) have agreed to the publication of this corrigendum; the other authors proved not to be contactable by the corresponding author.

